# *E. coli* cells advance into phase-separated (biofilm-simulating) extracellular polymeric substance containing DNA, HU, and lipopolysaccharide

**DOI:** 10.1128/jb.00309-24

**Published:** 2024-10-24

**Authors:** Archit Gupta, Purnananda Guptasarma

**Affiliations:** 1 Centre for Protein Science, Design and Engineering, Indian Institute of Science Education and Research (IISER) Mohali, Knowledge City, SAS Nagar, Punjab, India; 2 Department of Biological Sciences, Indian Institute of Science Education and Research (IISER) Mohali, Knowledge City, SAS Nagar, Punjab, India; Queen Mary University of London, London, United Kingdom

**Keywords:** DNA-binding proteins, biofilms, extracellular matrix

## Abstract

We have previously shown that the nucleoid-associated protein, HU, uses its DNA-binding surfaces to bind to bacterial outer-membrane lipopolysaccharide (LPS), causing HU to act as a glue aiding the adherence of DNA to bacteria, e.g., in biofilms. We have also previously shown that HU and DNA coacervate into a state of liquid-liquid phase separation (LLPS), within bacterial cells and also *in vitro*. Here, we show that HU and free LPS (which is ordinarily shed by bacteria) also condense into a state of phase separation. Coacervates of HU, DNA, and free LPS are less liquid-like than coacervates of HU and DNA. *Escherichia coli* cells bearing LPS on their surfaces are shown to adhere to (as well as advance into) coacervates of HU and DNA. HU appears to play a role, therefore, in maintaining both intracellular and extracellular states of phase separation with DNA that are compatible with LPS and LPS-bearing *E. coli,* with LPS determining the liquidity of the biofilm-simulating coacervate.

**IMPORTANCE:**

Understanding the constitution and behavior of biofilms is crucial to understanding how to deal with persistent biofilms. This study, together with other recent studies from our group, elucidates a novel aspect of the extracellular polymeric substance (EPS) of *Escherichia coli* biofilms, by creating a simulacrum of the EPS and then demonstrating that its formation involves liquid-liquid phase separation (LLPS) of HU, DNA, and lipopolysaccharide (LPS) components, with LPS determining the liquidity of this EPS simulacrum. The findings provide insight into the nature of biofilms and into how the interplay of HU, DNA, and LPS could modulate the structural integrity and functional dynamics of biofilms.

## INTRODUCTION

*Escherichia coli* forms conglomerations of cells known as biofilms. Within these, cells are held together by an extracellular polymeric substance (EPS) that is rich in nucleic acids (1). These nucleic acids include both RNA, and extracellular DNA (2, 3) bound to DNA-binding proteins (4). One abundant DNA-binding protein in the EPS is a non-sequence-specific, nucleoid-associated protein known as HU (5). The EPS in biofilms has been shown to undergo disintegration upon exposure to nucleases that degrade the extracellular DNA matrix, as well as upon exposure to antibodies cognate to the DNA-binding tips of DNABII proteins such as HU (6–8), suggesting that extracellular DNA somehow requires DNABII proteins like HU to be present in association with the DNA, for *E. coli* cells to be held together within the biofilm.

Previously, we have demonstrated that HU uses both its canonical and non-canonical DNA-binding surfaces to bind to lipopolysaccharide (LPS) present on the outer membranes of *E*. coli cells, with HU appearing to recognize hexose sugar-linked phosphate groups on the headgroup of LPS as a proxy for pentose sugar-linked phosphate groups on the backbone of DNA (9). Our demonstration that HU is a charge-neutralizing glue that allows the negatively charged surface of a bacterium to adhere to the negatively charged surface of DNA suggests mechanisms for both (i) the embedment of bacteria within a matrix of DNA and (ii) the generation of extracellular DNA to sustain the growth of biofilms through the occasional lysis of cells that tug at each other due to their mutual adherence to HU or HU-bound DNA. Notably, the explosive lysis of bacterial cells that appear to tug at each other has been observed experimentally and also shown to generate extracellular DNA (10, 11). Therefore, our identification of HU as a molecular glue assumes importance in answering questions both about (a) how negatively charged *E. coli* cells coexist with negatively charged DNA (9, 12) in biofilms, and also (b) about how extracellular DNA comes into existence in biofilms.

Independently, numerous experimental and computational (simulation-based) studies have suggested over the years that biofilms could be the result of phase transitions involving either liquid-liquid phase separation (LLPS) or the formation of a gel-like substance (13–15). Theoretically speaking, phase transitions involving the generation of varying degrees of liquid-like, gel-like, or solid-like behavior could most certainly hold benefits for the formation, maintenance, and differentiation of biofilms. This is because scope for differences between different regions of a biofilm (e.g., inner and outer regions or older and newer regions) could help a biofilm to become a complex collection of living cells, dead cells, and EPS derived from dead cells. Multiple hypotheses abound regarding how biofilms form through the phase separation of bacterial populations from their immediate environments (15). Given that a biofilm’s EPS is rich in DNA and DNA-binding proteins like HU, it is significant that we have recently demonstrated that HU and DNA crowd each other into liquid-liquid phase-separated (LLPS) states, both *in vivo* within bacterial genomic nucleoids and also *in vitro* (16). Thus, it appears plausible that biofilms are sustained through the cell lysis-based disgorgement of pre-formed biomolecular (HU-DNA) condensates derived from genomic nucleoids.

In other words, our previous work showing (i) that HU binds to LPS (9) and (ii) that HU and DNA crowd each other into LLPS states (16) seems to suggest that LLPS condensates of lysis-disgorged HU and DNA probably constitute the primary material of biofilms. Our work also suggests that bacterial cells remain embedded within this matrix through the binding of bacterial cell-surface LPS to the DNA-bound HU already present within extracellular LLPS condensates. This makes it necessary for us to explicitly examine whether *E. coli* cells show a preference for existing within condensates of HU and DNA (over existing in isotonic aqueous solutions), and also examine the role possibly played in such condensates by any free LPS that may be naturally shed by bacterial cells.

Given the above, we demonstrate below: (i) that HU phase-separates with free LPS, as it does with DNA, but that HU-LPS condensates are less liquid-like than HU-DNA condensates; (ii) that HU, DNA, and free LPS happen to coacervate to form mixed condensates that are less liquid-like than HU-DNA condensates; and (iii) that bacteria readily advance into HU-DNA condensates.

## RESULTS

### LPS and HU associate to undergo biomolecular condensation

We have previously shown that HU undergoes phase separation with DNA under physiological conditions (16, 17), using physiological concentrations of HU and DNA base pairs. Below, we demonstrate HU’s ability to undergo phase separation with LPS, under similar conditions, with one notable exception. With DNA, we had shown that HU forms condensates even in the complete absence of any macromolecular crowding agent such as polyethylene glycol (PEG); however, in order to reduce the amount of synthetic DNA used, we did use a nominal concentration of 2% PEG in all experiments carried out thereafter with HU and DNA. Here, with LPS, we found that the mutual crowding of HU-B and LPS is not as effective, presumably owing to the fact that each LPS molecule contains only two sugar-phosphate moieties for the binding of HU-B, unlike DNA which contains many more sugar-phosphate moieties for HU-B binding. Thus, there was no condensation in the absence of PEG. In the presence of a nominal PEG concentration of 4%, however, the addition of LPS to HU led to the formation of spherical biomolecular droplet-like condensates.

**Fig 1:**
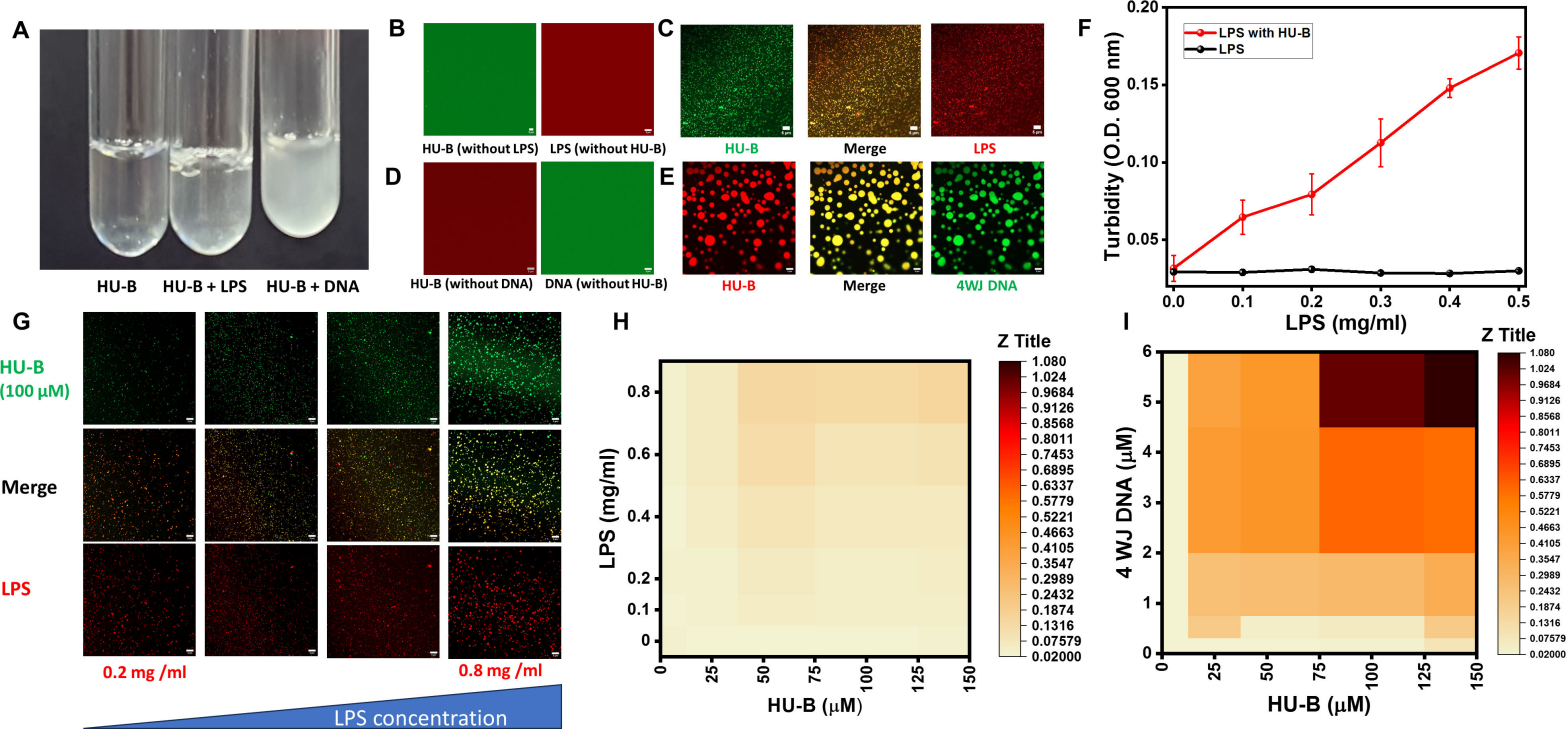
LPS and HU undergo biomolecular condensation. (**A**) Images showing turbidity created in HU on the addition of DNA or LPS. (B) Control confocal microscopy images of HU (labeled with Alexa Fluor 488) and LPS (labeled with Alexa Fluor 594). (C) Micrographs showing condensates created on mixing of HU-B (labeled with Alexa Fluor 488) and LPS (conjugated with Alexa Fluor 594). (D) Control confocal microscopy images of HU (labeled with Alexa Fluor 594) and DNA (conjugated with Fluorescein). (E) Micrographs showing condensates created on mixing of HU-B (labeled with Alexa Fluor 594) and DNA (conjugated with Fluorescein). (F) Turbidity-based examination of the effect of increasing LPS concentrations in the presence of HU (black curve) and in the presence of HU-B (red curve). (G) Micrographs of confocal microscopy showing the effect of increasing LPS (conjugated with Alexa Fluor 594). Phase diagrams of HU’s biomolecular condensation with (**H**) DNA and (**I**) LPS are shown with color intensities representing the turbidity of a particular reaction, and with the exact values mentioned under the Z-Title heading. All scale bars are of 5 µm. In panels A–E, concentration of HU-B is 100 µM, LPS is 0.4 mg per mL, and 4WJ DNA is 3 µM. In panel F, HU-B is present at 100 µM concentration in all reactions.

Figure 1A shows three tubes containing 100 µM HU-B in 150 mM salt (KCl), 20 mM Tris, and 4% PEG 6000, at pH 7.4, (i) in the complete absence of DNA or LPS, (ii) in the presence of LPS alone, and (iii) in the presence of cruciform 4-way junction (4WJ) DNA alone. For these experiments, similar wt/vol concentrations of LPS and DNA were used because the average molecular weight of LPS, which is 50–100 kDa (18–20), is similar to the molecular weight of 4WJ DNA (16), which is ~46.5 kDa. Cruciform DNA was used in these experiments both because HU-B displays a high affinity for cruciforms (21), which helps reduce the amounts of synthetic DNA used, and also because extracellular DNA has been reported to be rich in cruciform DNA (22). The tube containing HU-B alone displays no turbidity. Tubes containing either HU-B and DNA or HU-B and LPS show turbidity. Control experiments with tubes containing LPS (0.4 mg per mL) and 4WJ DNA (3 µM) are shown in Fig. S1, with both tubes showing no visible turbidity. The degree of turbidity in the tube containing HU and LPS is significantly lower than that seen in the tube containing HU-B and DNA. The LLPS nature of the entities giving rise to the turbidity was verified through fluorescence confocal microcopy. Figure 1B shows control confocal microscopic images of HU-B alone (100 µM), or LPS alone (0.4 mg/mL), revealing that no phase separation is seen in either instance, using the aforementioned buffer conditions. Figure 1C reveals that mixtures of HU-B and LPS form spherical condensates displaying both LPS-derived (red) and HU-derived (green) fluorescence. The Mander’s and Pearson’s coefficients of colocalization were calculated for HU-B and LPS (Fig. S2), and the colocalization was found to be significant, using all parameters used. The spherical morphology of the HU-LPS condensates was further validated using DIC imaging (Fig. S3). The presence of LPS inside the spherical condensates establishes that LPS acts not simply as a crowding agent that fails to enter condensates, but rather as an entity that actively engages with HU-B to undergo complex coacervation. This is clearly established by the overlap in the fluorescence signals derived from HU-B and LPS in the spherical condensates formed.

To compare the phase separation of HU-B with LPS and that of HU-B with DNA, we repeated the experiments shown in Fig. 1B and C, using DNA. Figure 1D shows control confocal microscopic images of HU-B alone (100 µM), and DNA alone (6 µM), with no evidence of any phase separation. Figure 1E confirms our earlier reports regarding the occurrence of phase separation through the mixing of HU-B and DNA, in a manner that leads to the formation of condensates that are both bigger and more well defined than the condensates formed by HU-B and LPS. We further verified the ability of LPS to modulate or increase the phase separation of HU-B, by increasing LPS concentrations and examining effects using both a turbidity-based assay (Fig. 1F) and fluorescence microscopy (Fig. 1G). Both experiments confirmed that higher concentrations of LPS elicit higher degrees of phase separation of HU-B although condensates remain consistently smaller than condensates formed by equivalent concentrations of HU-B and 4WJ-DNA. It may be noted, in passing, that HU exists in two isoforms in *E. coli*, HU-A and HU-B (5, 23, 24). Throughout this article, we have used only HU-B, owing to its higher relevance to stress-related conditions, e.g., starvation (25), applicable to biofilms (26); however, in Fig. S4, we also confirm the ability of the other HU isoform, HU-A, to phase separate with LPS and to form similar spherical biomolecular condensates that are rich in HU-A and LPS.

Next, we performed further comparisons of HU-LPS and HU-DNA condensates, using turbidity data as a proxy for the extent of phase separation, to construct phase diagrams of increasing HU-B concentration as a function of either increasing DNA concentration (Fig. 1H) or increasing LPS concentration (Fig. 1I), using similar regimes and ranges of wt/vol concentration. In these phase diagrams, LPS concentrations are annotated in mg/mL, and 4WJ DNA concentrations are annotated in μM since (as already mentioned) LPS has an average molecular weight of 75 KDa (18–20), causing 0.4 mg/mL LPS to be equivalent to approximately 5.33 µM LPS. On comparison of turbidities (represented as box color intensities in Fig. 1H and I) for every combination of HU and DNA/LPS concentration, mole for mole, 4WJ DNA is clearly seen to cause phase separation of HU to a much greater extent than LPS. We hold that this is due to there being more HU-B-binding sites on each copy of 4WJ DNA used (27), than on each copy of LPS, which contains only two sugar-phosphate moieties (9).

### Complex coacervation of LPS and DNA with HU

**Fig 2:**
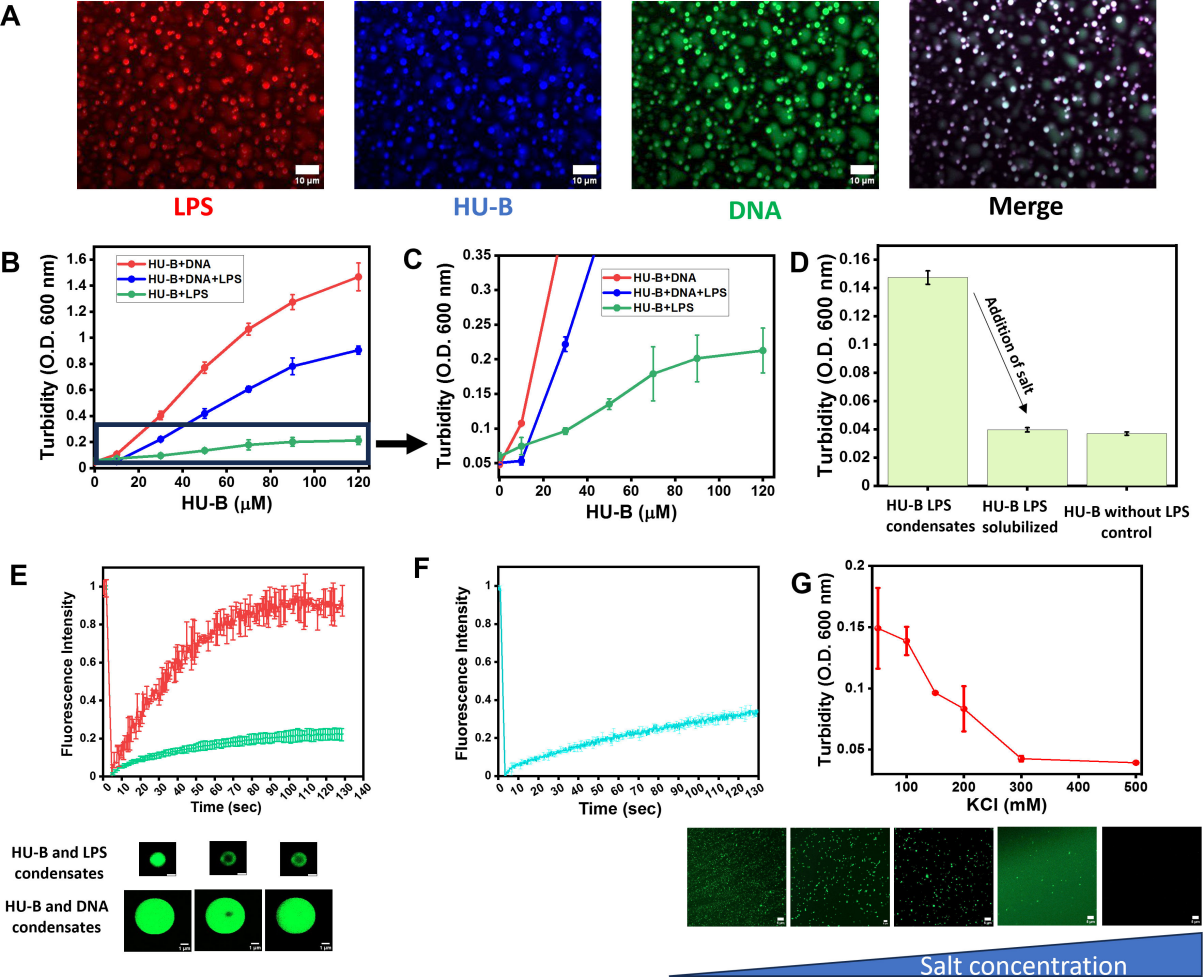
Effect of LPS on biomolecular condensation of HU and DNA, and the viscoelastic properties of condensates of HU with DNA or LPS. (A) Confocal microscopy images of complexcoacervation of HU (labeled with Alexa Fluor 647), LPS (labeled with Alexa Fluor 594), and conjugated with Fluorescein. (B) Turbidity-based analysis of effect of complex coacervation of HU, LPS, and DNA. (C) Expanded view of Fig. 2B. (D) Turbidity-based assay showing reversibility of HU-LPS condensates on the addition of salt. (E) Averaged kinetics of fluorescence recovery after photo-bleaching (FRAP) of Alexa 488-labeled HU-B in condensates of HU-B and DNA (red curve) and HU-B and LPS (green curve) with representative microscopy images of both experiments. (F) Averaged kinetics of fluorescence recovery after photo-bleaching (FRAP) of Alexa 488-labeled HU-B in condensates of HU-B and DNA spiked with LPS (final concentration 0.2 mg/mL). (G) Turbidity-based analysis of dependence on varying salt on HU-LPS biomolecular condensation. Microscopy images of the similar experiments are appended below. Figure 2A has 10 µm scale bars, 2E has 1 µm scale bars, and 2F has 5 µm scale bars.

We next examined the effect of the presence of free LPS upon the biomolecular condensation of HU-B and DNA. In Fig. 2A, LPS is demonstrated to be able to enter into condensates of HU-B and DNA to create complex coacervates that are rich in three molecular species; HU-B (blue), DNA (green), and LPS (red), all covalently labeled using different fluorophores. The calculation of colocalization coefficients shows that HU-B, LPS, and DNA display significant colocalization in the same condensates, as shown in Fig. S5, implying that the three are able to together to form biphasic heterotypic condensates. In quality (if not in actual relative quantities), this mixture mimics the composition of EPS, since the EPS is rich in DNA and HU (28), and also contains LPS shed from the surfaces of *E. coli* cells (29–31). In Fig. 2B, we see the results of a turbidity-based experiment assessing the phase separation induced by a combination of LPS and DNA. The red curve plots turbidity for HU-B phase separating with 6 µM DNA. The light green curve plots turbidity for HU-B with 0.4 mg/mL LPS (~5.33 µM). The blue curve plots turbidity for HU-B with both 6 µM DNA and 0.4 mg/mL (i.e., ~5.33 µM) LPS. The curves clearly show the absence of any additive effect. Rather they show a negative effect, i.e., the presence of both LPS and DNA does not increase the extent of phase separation, but rather decreases the extent of phase separation. We believe that this is because LPS and DNA are already known to compete for the same sites in HU-B, and because LPS is poorer in its ability to phase separate with HU, than DNA.

We also verified that the observed HU-LPS condensates are not “HU-LPS aggregates” by examining the reversibility of the formation of the condensates through the addition of salt, which would be expected to reduce the binding of HU to LPS. Figure 2D shows that the addition of 500 mM KCl to HU-LPS condensates formed in the presence of 150 mM KCl reverses the turbidity obtained initially. Figure S6 shows that condensates are no longer visible after the addition of 500 mM KCl.

### LPS can make HU condensates more gel like

There is another difference in the properties of HU-DNA and HU-LPS condensates besides the differences in their average sizes noted above. Unlike HU-DNA condensates, which display rapid FRAP, i.e., recovery of fluorescence after photobleaching of fluorophores attached to the components of the condensates (~90% recovery in less than 60 s), HU-LPS condensates show only nominal recovery of fluorescence after photobleaching. This suggests that HU-LPS condensates are more gel-like in nature and less liquid-like than HU-DNA condensates (see the green curve in Fig. 2E). Here, it must be noted that bleaching experiments with HU-LPS condensates that are typically very small posed significant experimental challenges. This caused us to resort to use of 10% PEG 6000 and 0.8 mg/mL LPS, specifically for these FRAP experiments, in order to try and make HU-LPS condensates somewhat bigger and more stable over time, without appearing to lose water to the bulk solvent (and so “dry up” rapidly). These attempts were made so that HU-LPS condensates could be subjected to photobleaching, with adequate scope remaining for the recovery of fluorescence after photobleaching, through diffusion of molecules from regions not covered by the laser, and not limited by the resolution of the experimental limits for specification of the region of interest targeted for bleaching.

Figure S7 shows, as a control experiment, that no morphological changes occur in condensates of HU-B and LPS with the use of high concentrations of PEG 6000, while the red curve in Fig. 2E shows FRAP data for condensates of HU-B and DNA under similar (control) PEG concentrations. This figure is shown while noting, in passing, that such conditions were not used in our previous paper reporting the discovery of the formation of LLPS condensates by HU and DNA (16). The control experiments establish that the high PEG concentrations used do not cause the observed lower liquidity since condensates of HU and DNA continue to show rapid FRAP at the very same high PEG concentrations used to obtain larger condensates of HU and LPS (10% PEG), in which almost no FRAP is observed.

Since the high PEG concentrations used in the control FRAP experiments do not necessarily simulate macromolecular crowding in natural biofilms, we next decided to examine whether the addition of moderate levels of LPS to HU-DNA condensates leads to the reduction of the liquidity of those condensates. Figure 2F shows that such “spiking” of condensates of HU-B and DNA by free LPS (which simulates the presence of LPS shed by bacteria, within biofilms of HU and DNA) causes a significant reduction in liquidity, as measured through FRAP. This suggests that regions of biofilms that have high concentrations of cell-free LPS, as well as the immediate environments of LPS-bearing bacteria that are bound to either free, or DNA-bound, HU in biofilms, could be associated with less liquid-like and more gel-like characteristics. It may be noted that the viscoelastic nature of biofilms has already become a subject of interest among some investigators and that modeling of the non-uniform viscoelastic nature of the extracellular matrix, over different regions of biofilms, has already been attempted (32, 33).

### As with HU-DNA, HU-LPS condensates are destructible through the destruction of electrostatic interactions by salt

We have shown in the above sections that condensates of HU-B and LPS differ from condensates of HU-B and DNA in certain respects despite the ability of the three species to form complex mixed coacervates. Here, we show that the molecular interactions driving the formation of the two types of condensates would appear to be similar. Just as HU-B’s interactions with DNA are governed by electrostatic forces, such forces also determine the formation of condensates of HU-B and LPS (16). Figure 2G shows that an increase in the concentration of KCl leads to a reduction in turbidity through a reduction of the formation of condensates of HU-B and LPS. The same conclusion was also derived from confocal fluorescence microscopic experiments shown in the same figure.

### LPS-bearing *E. coli* cells adhere to (and enter) condensates of HU-B and DNA

**Fig 3:**
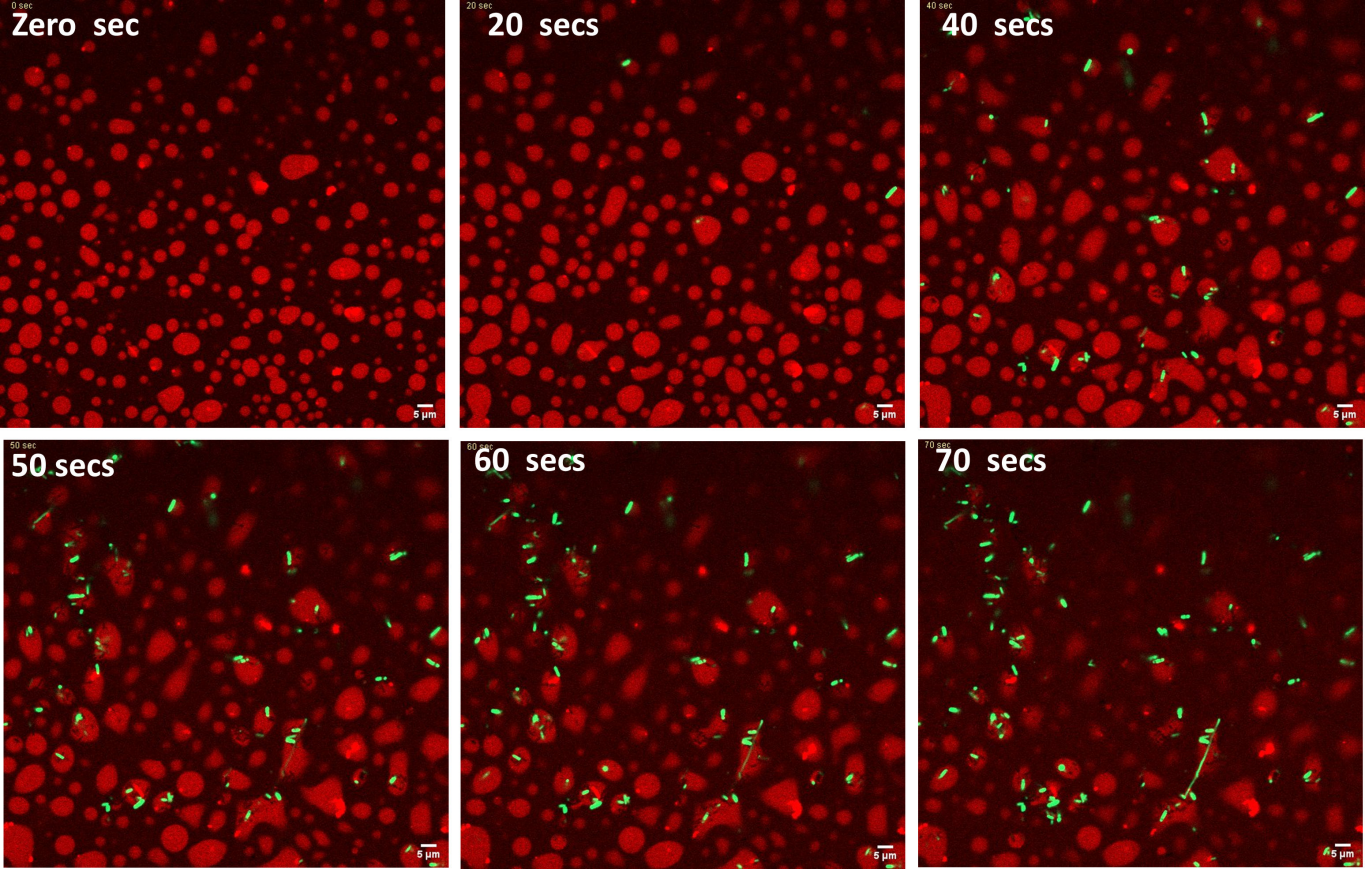
*E. coli* BL21 Star (DE3) pLysS cells adhere to HU-DNA condensates. A time series of confocal microscopy showing live *E. coli* cells preferring to adhere to HU-DNA condensates.

**Fig 4:**
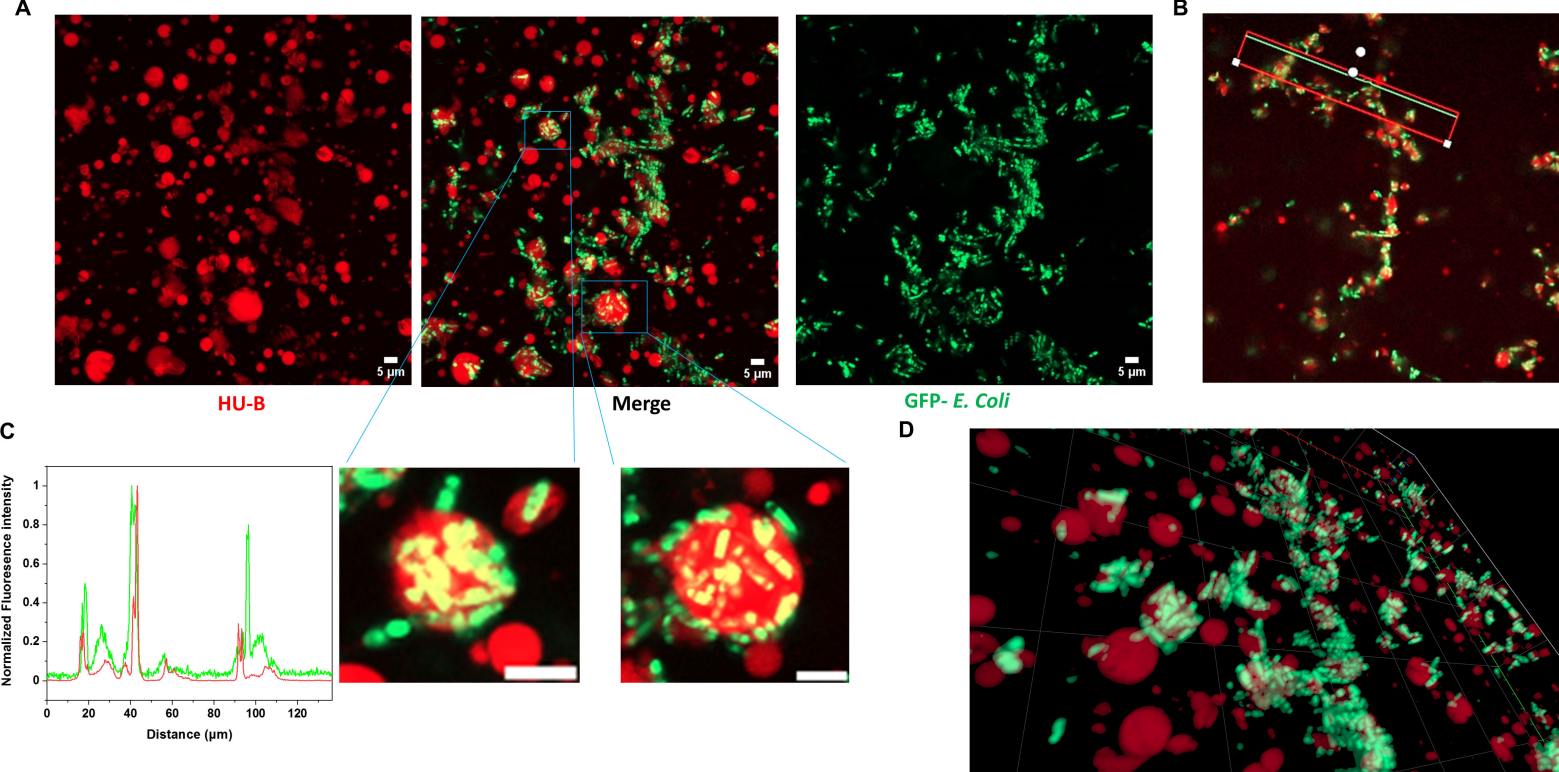
Arrangement of *E. coli* BL21 star (DE3) pLysS cells adhered to HU-DNA condensates. (A) Maximum intensity projections of Z-stacks from confocal microscopy of *E. coli* cells adhering to HU-DNA condensates. HU-B is labeled via Alexa Fluor 647, and *E. coli* cells are fluorescing green because of GFP expression. (B) Representation of a line that was drawn to calculate co-localization of condensates and *E. coli* cells. (C) Line profile showing intensities of HU condensates (red) and *E. coli* (green), using the line shown in Fig. 3B. (D) A zoomed 3-D image showing cells coating the HU- DNA condensates. All scale bars are of 5 µm.

In the above sections, we have demonstrated the compatibility of HU-B, DNA, and LPS in terms of their ability to form complex coacervates. Since HU-B binds to LPS through the same surfaces used by it to bind to DNA, as already shown earlier (9), it stands to reason that LPS-bearing *E. coli* cells may be anticipated to bind to “DNA-unoccupied” sites on multimeric phase separated forms of HU. We first initiated LLPS by addition of HU-B and DNA and added live *E. coli* cells expressing eGFP, on top of the sample, on the sample stage of the inverted microscope. In Fig. 3, we see that, over time, cells appear to be titrated toward condensates of HU-B and DNA. In Fig. 4A, maximum intensity projections of an experiment similar to the one shown in Fig. 3 are shown. In Fig. 3 and 4A, we can see that live GFP-expressing *E. coli* cells in stationary-phase adhere to (and enter) condensates of HU and DNA. We propose that the observed adherence, and entry, is the result of the titration of bacterial cells onto HU-B present on the surfaces of these condensates. We have already earlier shown that HU-B binds to the surfaces of *E. coli* cells and is able to cause the clumping of *E. coli* cells in both its free and DNA-bound forms (9).

Figure 4A, thus, presents results with a simulacrum of a biofilm, in which bacteria with negatively charged surfaces are able to become embedded within a matrix of DNA (also negatively charged) through the intercession of HU-B molecules that are able to act as a positively charged glue. The variation of fluorescence intensity along a line drawn through the bacteria-associated condensates (shown in Fig. 4B) is shown in Fig. 4C to demonstrate the colocalization of *E. coli* cells with condensates of HU-B and DNA, in that the presence of HU-B and DNA can be seen in the vicinity of most *E. coli* cells (although, of course, the presence of *E. coli* cannot be seen in the vicinity of all condensates formed by HU-B with DNA). In Fig. 4C, the green and red intensities of *E. coli* and HU-B, respectively, either overlap (for cells buried within condensates) or adjoin each other (for cells adhering to condensates). Figure 4D shows a zoomed-in section of a 3-D representation created through the overlapping of a stack of images collected along the *z*-axis, in a series of confocal microscopic experiments. Video S1 shows a video created out of merged channels of Z stacks from the experiment shown in Fig. 3, with every stack appearing as a frame in the video. This figure and the video confirm that bacterial cells adhere to (and enter) condensates of HU and DNA. In addition, Video S2 also shows frames of a single condensate as a function of viewing depth. In this video, it can be seen very clearly that some *E. coli* cells are attached to the condensate, while many *E. coli* cells are located in the interiors of the condensate.

## DISCUSSION

Biofilms are a way of life for microbes in the wild and also inside the bodies of other organisms. Inside organisms, biofilms that form on the surfaces of vessels carrying blood, or food, can potentially resist the entry of antibiotics and, thus, become hosts for populations of bacteria that can display proliferation after a course of antibiotics has concluded and also act as nurseries for the generation of antibiotic-resistant populations of mutant bacteria. Recent findings, such as those by Seviour et al., highlight the significance of surface proteins like Brosi-A1236 in *Candidatus Brocadia* in phase separation and cell-cell adhesion for biofilm formation (34). Staphylococcal Bap protein has also been shown to be able to form amyloids (after maturation of LLPS condensates) and act as an extracellular matrix scaffold in biofilms (35). Such findings prompted us to explore the role of LLPS in *E. coli* biofilm formation involving the nucleoid-associated protein, HU.

In this article, we demonstrate that liquid-liquid phase-separated condensates of a DNA-binding protein can act as a simulacrum of biofilms. We have used cellular concentrations of HU (36). DNA concentrations in the EPS of biofilms are known to fluctuate significantly, ranging from 1 to 40 mg/mL (37). To the best of our knowledge, LPS concentrations have never been determined experimentally; therefore, we have used a nominal concentration of 0.2 mg/mL in most experiments. This being said, it must be appreciated that the EPS inside a biofilm is highly dynamic and that metabolites inside biofilms are known to exist in different gradients and can be present at concentrations that are many folds higher at one point in the biofilm, than at another (38). Using such proxy concentrations, we show that HU, which is one of the most abundant and crucial protein components of the extracellular matrix in bacterial biofilms (6, 39), undergoes complex coacervation with DNA and/or LPS to create condensates whose liquidity is determined by LPS content, with LPS acting like a mimic of DNA and binding to DNA-binding sites on HU through electrostatic interactions. It is known that *E. coli* cells shed LPS into the extracellular environment. We show, using fluorescence recovery data after photobleaching, that LPS makes phase-separated condensates of HU-B and DNA more gel-like, from their originally liquid-liquid phase-separated states. Presumably, this allows cells to stick to surfaces with high fluid dynamics and turbulence, such as wet rocks, gut lining, and the inner surfaces of blood vessels. Taking together our previous work and this article, we show that HU probably plays a central role in phase separating with DNA both within bacterial cells (to form a phase-separated genomic nucleoid) and outside bacterial cells (to form the body of a biofilm). The phase separation of the proteinaceous and extracellular polymeric substances in a biofilm provides benefits such as the exclusion of nucleases, or proteases, and other molecules (such as antibiotics) that could affect cell viability, by slowing down diffusion in a manner modulated by LPS concentration.

LPS is known to be capable of existing in a phase-separated state in the outer membrane of *E. coli* and to create LPS raft-like structures that are most likely involved in immunoregulation (40). There exists a possibility that phase-separated LPS on bacterial cell surfaces facilitates the interaction of HU-DNA-LPS in the EPS and allows the adhesion of cells to each other and to the EPS. We show that *E. coli* cells move into condensates of HU-B and DNA, presumably because HU-B binds to cell-displayed LPS and also because condensates contain a higher concentration of HU-B than the bulk solvent, thus allowing HU-DNA condensates to essentially act like kernels for initiating biofilm formation. More challenging studies will be required to understand how the physiological concentrations of LPS in biofilms (which remain difficult to determine) might influence bacterial interactions and bacterial cell density. In conclusion, in this article, we suggest that biofilms may benefit from the phase separation of DNA with DNA-binding proteins. We also show that the addition of LPS to phase-separated states of DNA and HU can make the EPS more gel-like, in a manner that could aid in cellular embedment.

## MATERIALS AND METHODS

### Materials

#### 4WJ DNA

4WJ DNA was generated *in vitro* by annealing of four different oligos (purchased from Merck) in equimolar amounts to create a Holliday junction-like (cruciform) structure. The nucleotide sequences of four oligos used were as follows:

Strand 1–5′-CCCTATAACCCCTGCATTGAATTCCAGTCTGATAA-3′,

Strand 2–5′-GTAGTCGTGATAGGTGCAGGGGTTATAGGG-3′,

Strand 3–5′-AACAGTAGCTCTTATTCGAGCTCGCGCCCTATCACGACTA-3′,

Strand 4–5′-TTTATCAGACTGGAATTCAAGCGCGAGCTCGAATAAGAGCTACTGT-3′.

4WJ DNA was used at a concentration of 3 µM unless stated otherwise

#### Labeled 4WJ DNA

To prepare fluorescently labeled 4WJ DNA we purchased (from Merck) the aforementioned strand 2 with a 3′ FAM moiety covalently attached to it. Therefore, the final sequence was 5′ GTAGTCGTGATAGGTGCAGGGGTTATAGGG-FAM-3′. After assembling with the three remaining strands, we effectively had one FAM molecule per 4WJ DNA molecule.

#### Lipopolysaccharide

Purified lipopolysaccharide originating from *E. coli* O111:B4 was purchased from Merck (Catalog number L2630).

#### E. coli cells used

For imaging experiments stationary phase *E. coli* cells of the strain BL21 Star (DE3) pLysS were used. The cells were made to fluoresce green by the expression of eGFP protein that was being expressed from pET23a vector by IPTG induction. Cells were induced at an OD of 0.6 and then were cultured further at 37°C till they reached an O.D. of about 2. Cells were washed in a PBS buffer of pH 7.4 before imaging.

### Methods

#### Turbidity assays

All components were mixed on the bench and then immediately transferred to a 96-well plate for absorption measurements in a BMG Labtech POLARstar Omega plate reader. Absorption readings were taken at 600 nm at 37°C.

#### Reversibility of HU and LPS condensates

Phase separation was induced, as usual, by adding LPS to HU. The formation of condensates was verified by measuring turbidity and by visualization using confocal fluorescence microscopy. To these preformed condensates, KCl was added from a stock of 3M, and the dissolution of condensates was verified by the same assays as described above.

#### Microscopy

Confocal fluorescence microscopic imaging was performed using a ZEISS LSM 980 Elyra 7 super-resolution Microscope using a × 63 oil-immersion objective with a numerical aperture of 1.4, coupled with a monochrome-cooled high-resolution AxioCamMRm Rev. 3 FireWire(D) camera. Samples for microscopy were prepared by mixing on the bench and were imaged immediately thereafter, using makeshift chamber slides. For samples with proteins, fluorescently labeled proteins were spiked into the unlabeled proteins; spiking was done with 1%; 99% of the protein was accounted for by unlabeled protein with no cysteine mutation. All images were analyzed using Fiji ImageJ.

#### Protein purification and concentration estimation

Protein expression and concentration protocols have been detailed in previous articles (9, 16). Briefly, pQE-30 plasmids (Qiagen) containing the gene encoding HU-B F47W were transformed into XL1-Blue cells, and expressed protein was purified using Ni-NTA chromatography, followed by cation exchange chromatography. The protein used in this article was a point mutant (HU-B F47W) of the wild-type HU-B protein. HU-BF7W was used because wild-type HU has no tryptophan in it, for reasons elaborated earlier (41). Without tryptophan in its sequence, it is difficult to calculate protein concentrations accurately; therefore, we have used the mutant which we have shown to not cause effect on DNA binding or phase separation ability of HU (9, 16).

#### FRAP assay

FRAP assays were carried out using a Zeiss LSM 980 microscope. In all cases, HU-B was bleached using a 488 nm laser line. For bleaching, 100% intensity of laser was used and 100 iterations were made in a circle of 0.5 µm diameter. Experiments were done in triplicates, and fluorescence intensities were plotted after normalization.

#### Fluorescent labeling

LPS with covalently conjugated Alexa Flour 594 was purchased from Merck (cat. No. L23353). A cysteine mutant of HU, described previously (16), was used to label HU with Alexa fluor 647 C_5_ Maleimide or Alexa Fluor 488 C_5_ Maleimide. The procedure for labeling involved overnight incubation of 100 µM HU-B with 300 µM of TCEP (Tris(2-carboxyethyl) phosphine hydrochloride) and 200 µM of dye. Ultracentrifugation was done to remove free dye.

#### Imaging of cells adhering to condensates of HU and DNA

Condensates of HU-B and DNA were made *in vitro* with spiking of either Alexa 594-labeled HU or Alexa Fluor 647 labeled HU. The condensates were then visualized using LSM 980 confocal microscope. On top of the coverslip, containing HU-B and DNA condensates, stationary phase *E. coli* cells were added. An aliquot of the mixture was visualized immediately, and 3-D rendition of the z stacks was prepared using Zen blue edition software. The line profile for fluorescence intensities was also prepared using Zen blue edition software, and extracted raw values were plotted using origin pro 2020b.

#### Colocalization image analysis

Images were loaded into ImageJ, followed by the application of max entropy thresholding. The Pearson’s correlation coefficient and Mander’s overlap coefficients (MI and M2) were then computed using the JaCOP plugin.

#### Statistical analysis

All experiments were done in triplicates. The data for mean values were plotted along with the standard deviation. The statistical significance analysis was performed using one-way ANOVA analysis of variance test, using the software Origin Pro 2021b. All data analysis, data fitting, and data plotting were performed using Origin Pro 2021b.

## Data Availability

All the data for this manuscript are contained in the article above and the supplementary information file.

## SUPPLEMENTAL MATERIAL

Supplemental material jb.00309-24-s0001.docx Fig. S1 to S7; Videos SV1 and SV2 legends.

Supplemental SV1 and SV2 jb.00309-24-s0002.mp4 Videos showing *E. coli* cells.

ASM does not own the copyrights to Supplemental Material that may be linked to, or accessed through, an article. The authors have granted ASM a non-exclusive, world-wide license to publish the Supplemental Material files. Please contact the corresponding author directly for reuse.
