# Supplementary material for: E. coli cells advance into phase-separated (biofilm-simulating) extracellular polymeric substance containing DNA, HU, and lipopolysaccharide
Source: J Bacteriol. 2024 Oct 24;206(11):e00309-24. doi: 10.1128/jb.00309-24 (PMC11580401; doi:10.1128/jb.00309-24)
Supplement: Supplemental material — Fig. S1 to S7; Videos SV1 and SV2 legends. [file jb.00309-24-s0001.docx]

**SUPPLEMENTARY FIGURES FILE**

For

*E. coli* cells advance into phase-separated (biofilm-

simulating) extracellular polymeric substance containing DNA, HU, and lipopolysaccharide

Archit Gupta^1,2,*^ and Purnananda Guptasarma^1,2,*^

^1^Centre for Protein Science, Design and Engineering ^2^Department of Biological Sciences; Indian Institute of Science Education and Research (IISER) Mohali, Knowledge City, Sector 81, SAS Nagar, Punjab 140306, India

^*^ Author for correspondence


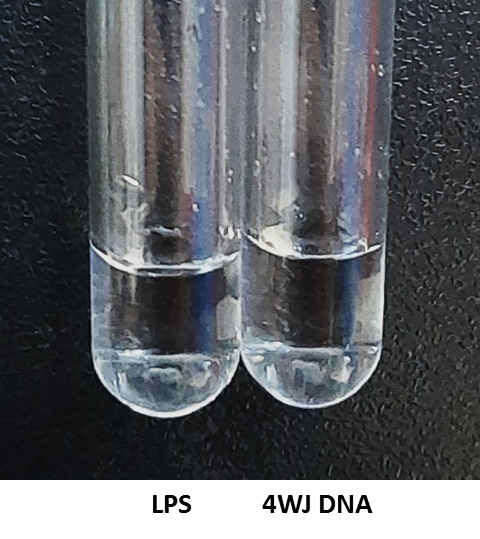


Supplementary Fig. S1- Glass tubes containing LPS (left) and DNA (right) showing no turbidity in absence of HU-B and in presence of 20 mM Tris, pH 7.4, 150 mM KCl and 4 % PEG 6000.

**1**

**0.8**

**Colocalization coefficients of HU and LPS**

**0.6**

**0.4**

**0.2**

**0**

**Pearson M1 M2**

Supplementary Fig. S2- Pearson’s colocalization coefficient (PCC) and Mander’s (M1/M2) colocalization coefficients of HU-B with LPS. M1 represents the fraction of HU-B in condensates positive for LPS and M2 being the fraction of LPS in condensates positive for HU-B


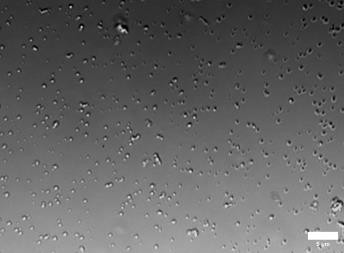


**DIC**


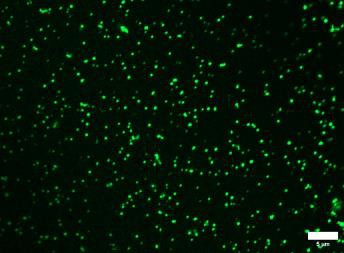

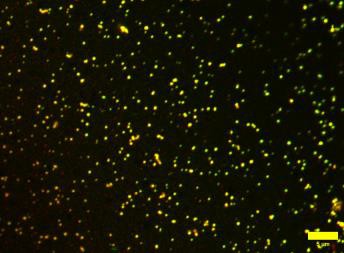

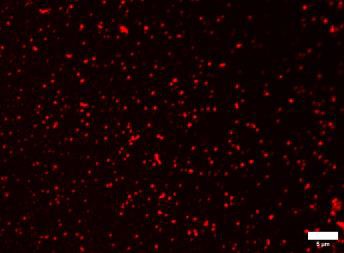


**HU-B Merge LPS**

Supplementary Fig. S3- DIC image of HU-LPS condensates. Fluorescence images are also attached to verify that LPS is also present in the condensates. HU-B (labelled with Alexa Fluor 488) and LPS (conjugated with Alexa Fluor 594).


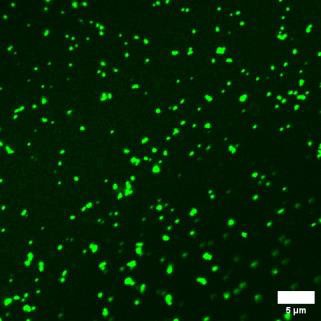

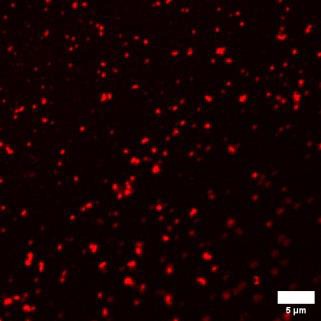
**A**


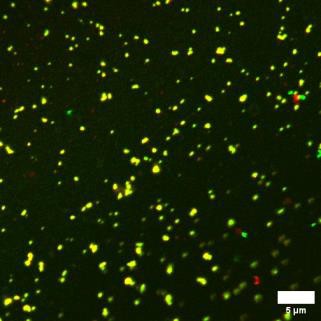


**B**

**0.18**

**~~***~~**

**Turbidity (O.D. 600 nm)**

**0.16**

**0.14**

**0.12**

**0.1**

**0.08**

**0.06**

**0.04**

**0.02**

**0**

**HU-A**

**HU-A with LPS**

**LPS**

Supplementary Figure S4- LPS can cause biomolecular condensation of HU-A, similar to HU-B. (A) Microscopy-based images showing condensation by HU-A (labelled with Alexa Fluor 488) and LPS (conjugated with Alexa Fluor 594). (B) Turbidity-based examination of increased turbidity of HU-A in presence of LPS, analyzed for triplicates using one-way ANOVA, with *** indicating p < 0.001.

**A B**

**1 1**

**Colocalization coefficients of HU and LPS**

**Colocalization coefficients of LPS and DNA**

**0.8 0.8**

**0.6 0.6**

**0.4 0.4**

**0.2 0.2**

**0**

**Pearson M1 M2**

**C**

**0**

**Pearson M1 M2**

**1.0**

**Colocalization coefficients of HU and DNA**

**0.8**

**0.6**

**0.4**

**0.2**

**0.0**

**Pearson M1 M2**

Supplementary Figure S5. (A) Pearson’s colocalization coefficient (PCC) and Mander’s (M1/M2) colocalization coefficients of HU-B with LPS. M1 represents the fraction of HU-B in condensates positive for LPS and M2 being the fraction of LPS in condensates positive for HU-B. (B)Pearson’s colocalization coefficient (PCC) and Mander’s (M1/M2) colocalization coefficients of LPS with DNA. M1 represents the fraction of LPS in condensates positive for DNA and M2 being the fraction of DNA in condensates positive for LPS. (C) Pearson’s colocalization coefficient (PCC) and Mander’s (M1/M2) colocalization coefficients of HU-B with DNA. M1 represents the fraction of HU-B in condensates positive for DNA and M2 being the fraction of DNA in condensates positive for HU-B.


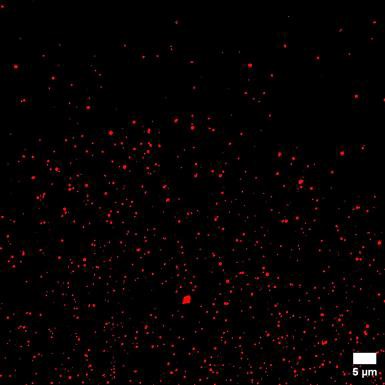

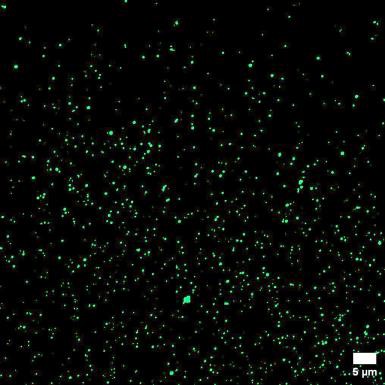


**On addition of 500 mM KCl, condensates disappear within 10 mins**


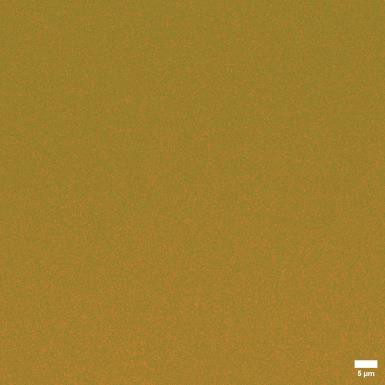

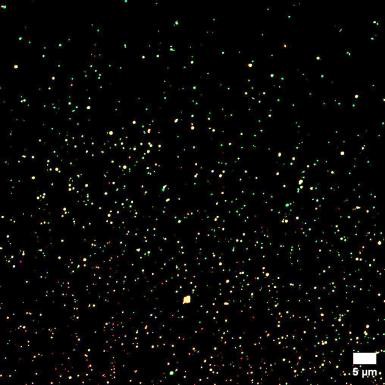


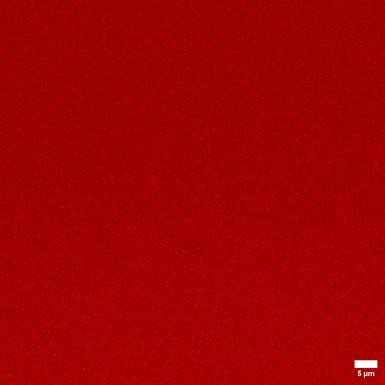

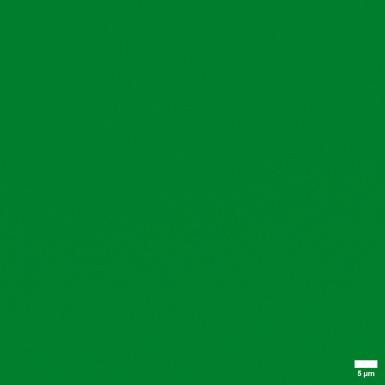


Supplementary Figure S6- Microscopy based examination of disappearance of condensates on addition of salt to the preformed condensates. HU-B is labelled with Alexa Fluor 488 and LPS is conjugated with Alexa Fluor 594. Scale bar is 5 µm in all panels.


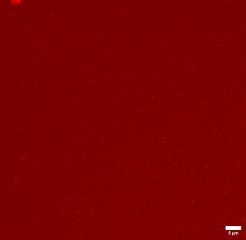

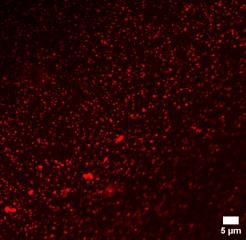

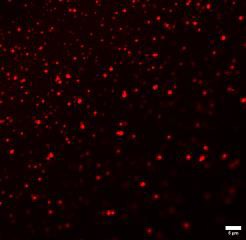

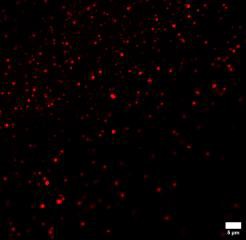
LPS


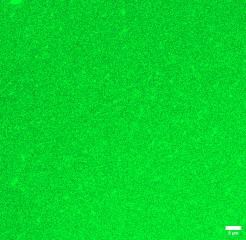

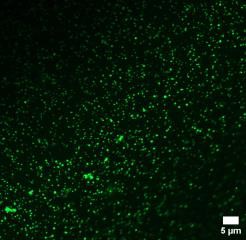

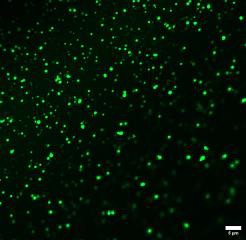

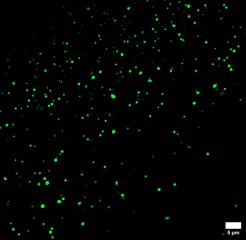
HU-B


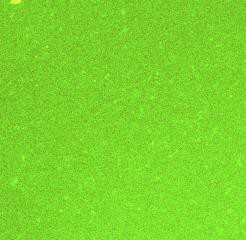

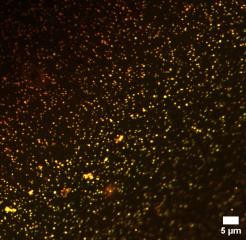

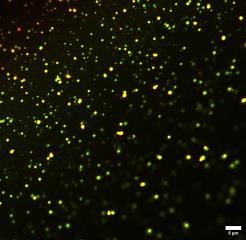

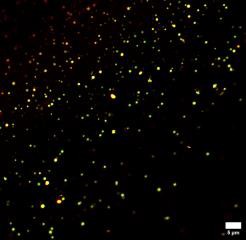
Merge

Zero (%) 4 (%) 6 (%) 10 (%)

PEG concentration

Supplementary Figure 7 - Microscopy images showing the effect of increasing concentrations of PEG 6000 from 0% to 10% W/V. HU-B is labelled with Alexa Fluor 488 and LPS is conjugated with Alexa Fluor 594. Scale bar is 5 µm in all panels.

**Legends to the Supplementary movies**

Supplementary video 1: This video is derived from the same that is described in Figure 4A and Figure 4D in the main manuscript. Whereas Figure 4A showed a maximal intensity projection integrating all Z planes, and Figure 4D shows a pseudo-3D representation, this video shows fluorescence for the entire sample, as individual z- stacks, progressing through all planes from top to bottom, with bacteria showing up in green and condensates of HU and DNA showing up in red. The sale bar is for 5 microns.

Supplementary video 2: This video is derived from the same that is described in Figure 4A and Figure 4D in the main manuscript. Whereas Figure 4A showed a maximal intensity projection integrating all Z planes, and Figure 4D shows a pseudo-3D representation, this video shows fluorescence for an individual condensate, as individual z- stacks, progressing through all planes from top to bottom, with bacteria showing up in green and condensates of HU and DNA showing up in red. This video presents that bacteria both adhere to the condensates, and are also present within the condensates. The sale bar is for 5 microns.
